# Assessment of the adequacy of mobile applications for disaster reduction

**DOI:** 10.1007/s10668-021-01697-2

**Published:** 2021-09-06

**Authors:** Lucía Navarro de Corcuera, María del Mar Barbero-Barrera, Ana Campos Hidalgo, Jorge Recio Martínez

**Affiliations:** 1 ICHaB-ETSAM. ETSAM, Arcoíris NGO, Universidad Politécnica de Madrid, Avenida Juan de Herrera 4, 28040 Madrid, Spain; 2 Department Construction and Technology in Architecture. ICHaB-ETSAM. ETSAM, Universidad Politécnica de Madrid, Avenida Juan de Herrera 4, 28040 Madrid, Spain; 3 Universidad Politécnica de Madrid, Avenida Juan de Herrera 4, 28040 Madrid, Spain; 4 Universidad Politécnica de Madrid (Spain), Avenida Juan de Herrera 4, 28040 Madrid, Spain

**Keywords:** Disaster Risk Management, Information and Communication Technologies, Mobile applications, Natural hazards

## Abstract

Natural events continue to take a heavy toll on human lives. Added to this are the challenge of dynamic at-risk settings, uncertainty, and increasing threats, which demand holistic, flexible, and quickly adaptable solutions. In this context, mobile applications are strongly emerging as communication tools that can assist in disaster reduction. Yet, these have not been sufficiently evaluated. In view of this, the aim of this research is to evaluate the adequacy of mobile applications in disaster risk reduction in reference to some of the deadliest natural events. To this purpose, a two-part methodology is developed. Firstly, a random sample of applications is evaluated and contrasted with the literature. Secondly, the viability of mobile applications is determined based on the Digital Application Potential Index proposed by the authors, cross-referenced in Geographical Information Systems with the WorldRiskIndex. The results show that most mobile applications limit their coverage range to only one stage of Disaster Risk Management (DRM) and one type of hazard event, failing to address systemic risk and hampering the scale-up of humanitarian response. For these to become adequate and wide-reaching, strong policies to promote reliability, transparency, and citizen empowerment would be required. The policies establishing the use of mobile applications as a viable tool for DRM must consider reducing the prices of internet connectivity while increasing educational levels, on top of language translation. At this point, the adoption of mobile applications is unable to ensure DRM communication, especially in countries with higher-risk levels, requiring these to be complemented with auxiliary tools.

**Graphic abstract:**

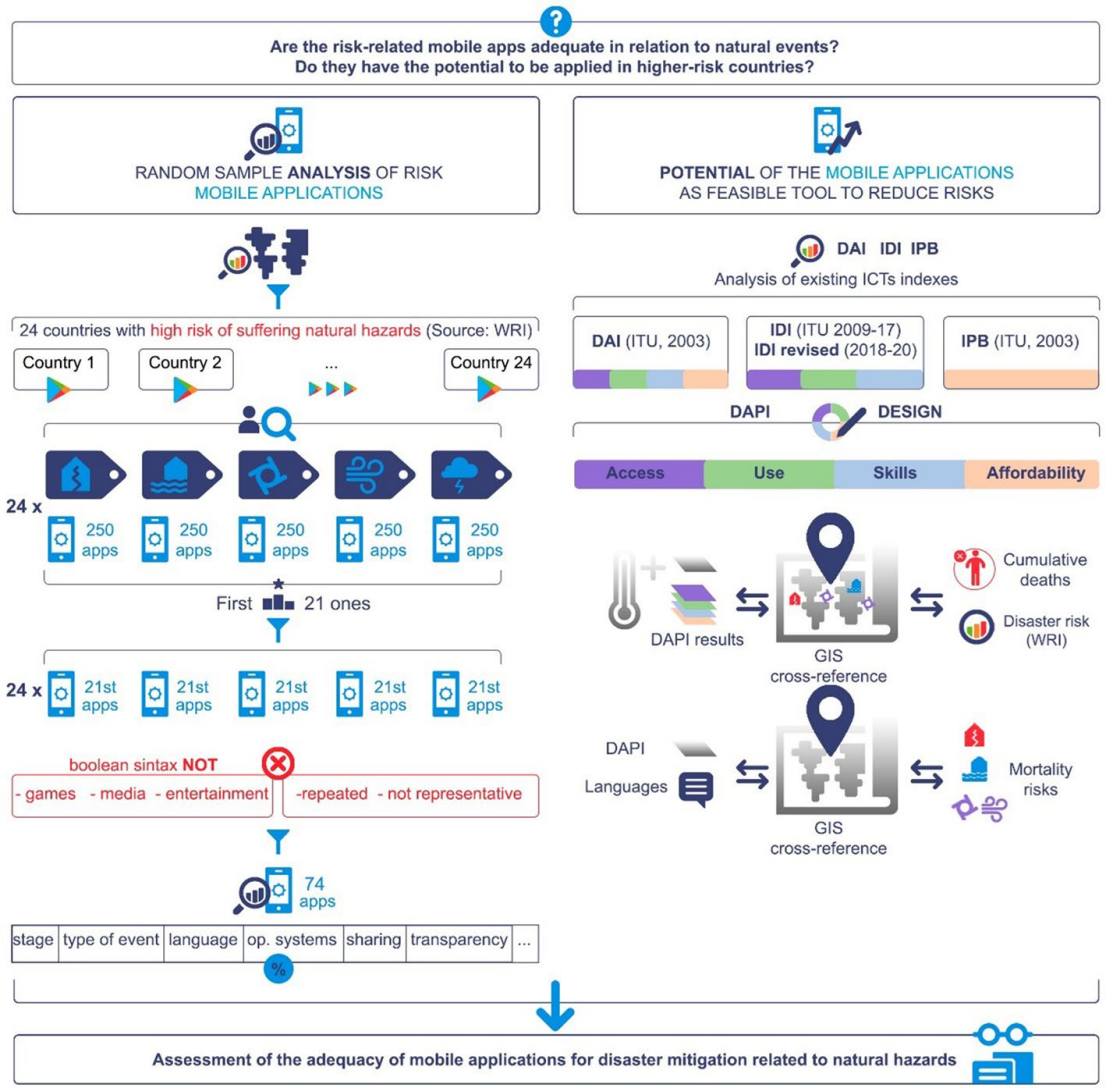

## Introduction

A preliminary note on terminology: It is important to understand that the authors have intentionally avoided using the term natural disasters. Despite its colloquial use, it is incorrect. A natural event becomes a disaster when multiple factors converge that are not natural at all (World Bank & United Nations, 2010). Therefore, instead of ‘natural’ disasters, we will refer to disasters related to natural events.

### Disasters associated with natural hazards

According to the 2020 risk report, extreme weather events and disasters associated with natural hazards are in the top three long-term global risks in terms of likelihood for the third consecutive year (WEF, 2020). These are amplified by the emergence of growing inequality amidst socio-natural situations, climate change, and climate action failure (CRED & USAID, 2020; Evans, 2019; Guha-Sapir et al., 2015; UNDRR, 2019). The number of deaths worldwide due to natural events is highly variable from year to year, averaging about 60,500 people in the last 25 years (pre-COVID-19 data). However, when taking injured, affected, and homeless into account, this amount rises to 5 billion (CRED & USAID, 2020; Guha-Sapir et al., 2015), to which about 24 million annual migrants must be added (UNDRR, 2019).

The most deadly natural hazards between 1995 and 2020 (CRED & USAID, 2020; Guha-Sapir et al., 2015) were earthquakes, cyclones, and floods. Earthquakes caused more than 760,000 deaths (49% of total deaths), followed by storms (including hurricanes, cyclones, and storm surges) with more than 250,000 fatalities (16% of total deaths, of which cyclones accounted for 15%). Finally, floods had the farthest reaching impact, affecting 2.5 billion people (Table 1) and resulting in 11% of total deaths (over 170,000 people).

**Table 1:**
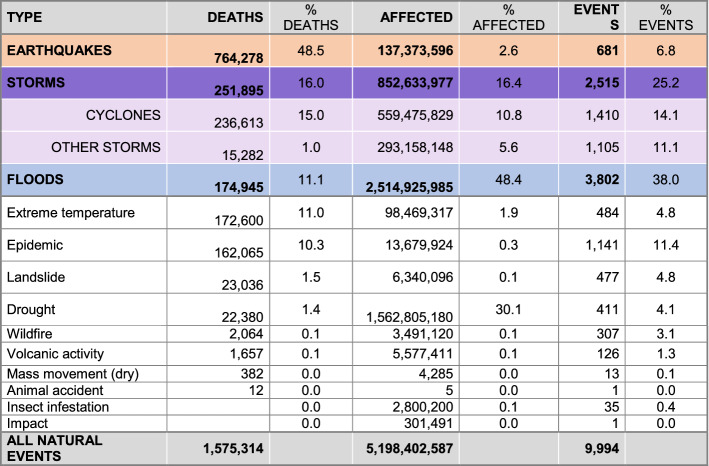
Number of events, deaths (including missing people), and affected people (sum of injured, affected, and homeless) by type of natural event from 1995 to 2020

Calculations from (CRED & USAID, 2020; Guha-Sapir et al., 2015)

**Fig. 1:**
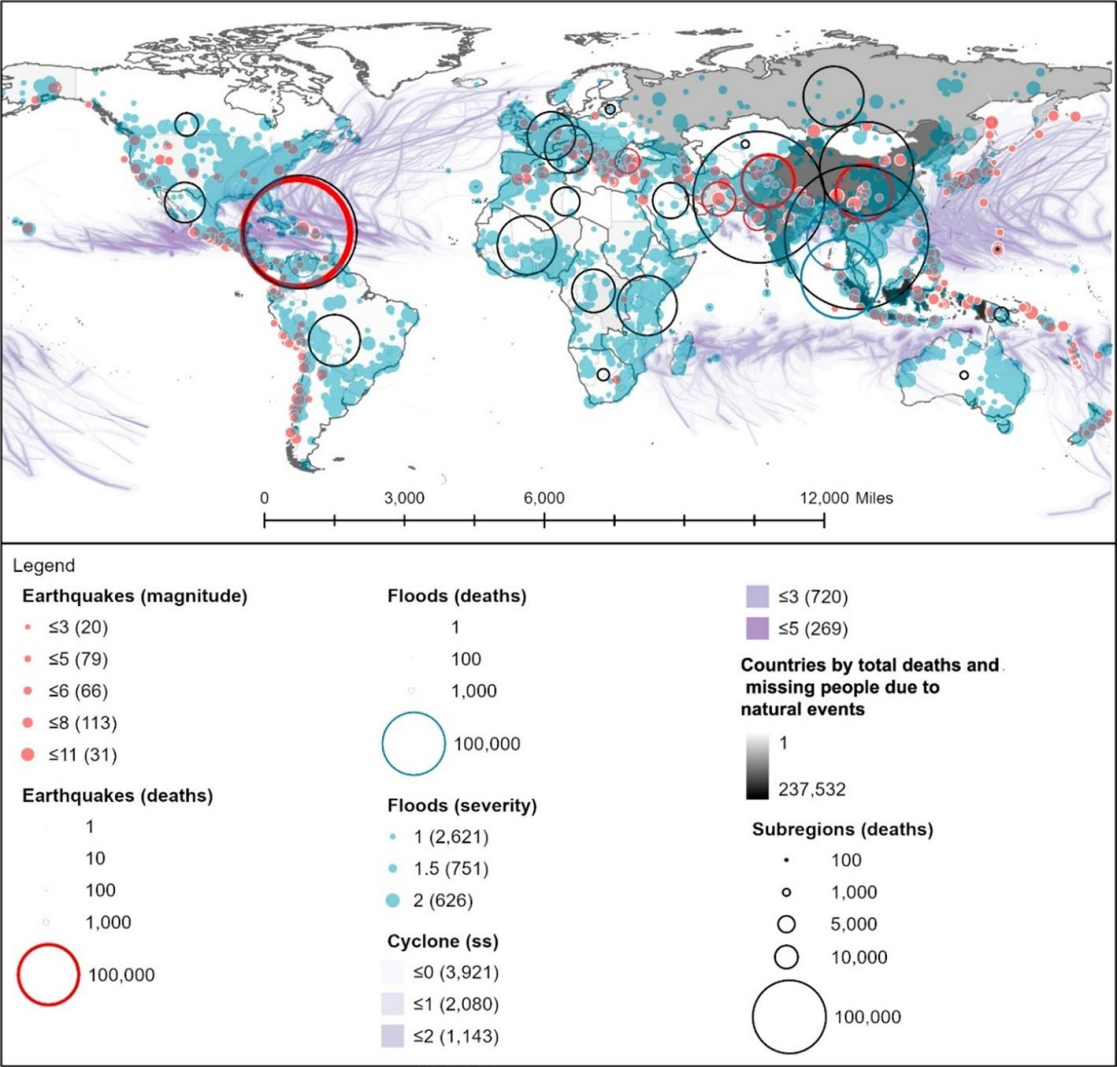
Location, intensity, and deaths linked to historical floods, earthquakes, and cyclones, based on: flooding events by severity (Severity Classification according to the Dartmouth Flood Observatory (DFO). The assessment is conducted on a scale from 1 to 2. Floods are then divided into three classes. Class 1: large flood events: significant damage to structures or agriculture; fatalities; and, or 1 to 2 decades-long reported intervals from previous similar event. Class 1.5: very large events: estimated recurrence interval composed of 20 to 100 years, and, or a local recurrence interval of 1 to 2 decades that affects a large geographic region (> 5000 km2). Class 2: Extreme events: with an estimated recurrence interval of more than 100 years) and deaths (Brakenridge, n.d.; Dartmouth Flood Observatory, 2020); major earthquakes (with magnitudes higher than 5.0) by magnitude and deaths by ANSS (ANSS—Advanced National Seismic System, 2020). Conversion to shapefile in ArcGIS and extraction of the 1995–2000 period; tropical cyclones windspeed buffers according to the Saffir-Simpson category scale. UNEP/DEWA/GRID-Europe (Global Risk Data Platform, 2020). Extraction of the 1995–2000 period; shapefile countries: Esri, Garmin, CIA World Factbook (References:: Regional and World Maps—The World Factbook—Central Intelligence Agency, 2020). The deaths and missing persons attributed to disasters were linked by country ISO code from the Emergency Events Database (EM-DAT); the subregional and regional shapefile and data were created by dissolving countries according to the region codes in the Standard country or area codes for statistical use (M49) and based on the United Nations Development Programme Statistical Division (Regional Groupings—SDG Indicators, 2020). The sum of total deaths registered by country ISO code EM-DAT was added to the shapefile

The cumulative number of deaths between 1995 and 2020 is graphically displayed in Fig. 1, following a light-to-dark scale according to severity. As can be observed, floods are the most widespread climate event, highlighting climate change effects (Chang & Franczyk, 2008; Economy, 2018), while storms are the most deadly natural phenomenon (Wahlstrom & Guha-Sapir, 2015), with a higher prevalence in Eastern Asia. The latter is also an earthquake-prone area, together with the West coast of America, which exhibits the leading number of deaths.^1^

### A systemic and complexity approach to disaster risk management (DRM): flexible and quick strategies

**Fig. 2:**
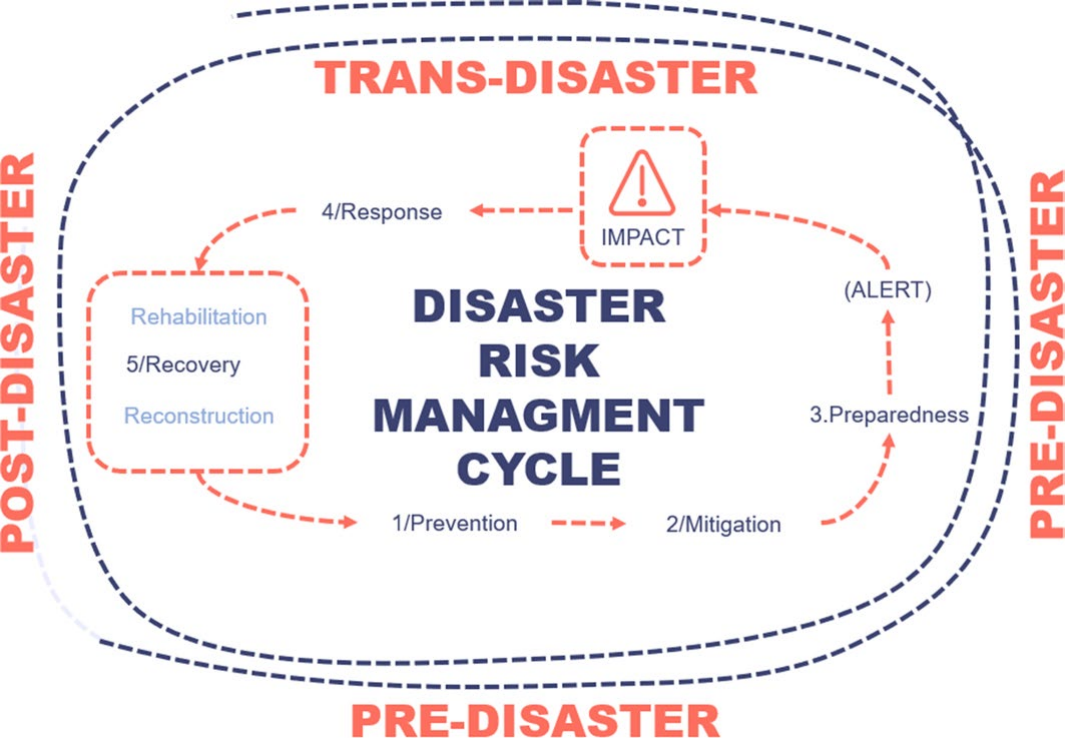
DRM Stages. Preparedness by the authors and adapted from (Arce et al., 2012; Coppola, 2011; Foresti, 2015; Ludwig & MATTEDI, 2018; Uitto & Shaw, 2016)

The concept of risk is intertwined with that of systemic epistemology, in that it entails a multi-dimensional perspective, scale, and impact (UNDRR, 2019). A hazard turns into a disaster when the magnitude and impact of the event lead to vulnerable states, where the capacity of subjects to cope with the event (resilience) and to repair what has been damaged and reconstruct itself (self-poiesis) (Cardona Arboleda, 2008) has been compromised. The relationship between complexity and risk, together with social and environmental aspects, emerges from the global framework for DRM (UNDRR, 1994), which includes prevention, mitigation, and preparedness (UN, 2015; Wahlstrom & Guha-Sapir, 2015). To achieve a comprehensive DRM, five main steps are considered at all stages (Fig. 2): 1. prevention; 2. mitigation; 3. preparedness; 4. response; 5. recovery (Arce et al., 2012; Coppola, 2011; Uitto & Shaw, 2016).^2^

To address the complexity of risk, including change and uncertainty, the challenge lies in designing holistic, flexible, and effective plans, as well as strategies that set the path for a quick response and the ability to adapt solutions in real time (UNDRR, 2019). For this purpose, clear communication is essential at all stages of DRM (Giroux et al., 2009; Victor, 2014). In fact, the effectiveness and quality of communication with the local population, before and after the phenomena, is a key point for mitigating disasters related to natural hazards.

### Are Information and Communication Technologies (ICTs) the solution for DRM?

Technological tools could constitute an alternative to facilitate efficient and rapid communication (Verrucci et al., 2016), as these have become increasingly widespread among the population. Mobiles, in particular, would be a viable tool, considering that about 70% of the global population is projected to be subscribed to mobile services (about 5.8 billion people) in 2025 (Association, 2020) and that most of the world’s population lives within reach of a mobile cellular signal (97%) (ICT et al., 2019).

In the last decade, research on the potential of ICTs in DRM has been carried out (Cioca et al., 2009; Ludwig & MATTEDI, 2018; Reddick, 2011; Wang et al., 2016), covering a wide range of uses, such as SMS (Cioca et al., 2009; Homier et al., 2018), television broadcast (Grassau et al., 2019; Segura et al., 2015; Wahyu et al., 2012), radio (Cardoso et al., 2014), social network (Doktor & Giroux, 2011; Dunn Cavelty & Giroux, 2011; Norris, 2017; Peterson et al., 2019; Rogstadius et al., 2011), e.g. Twitter (Hong et al., 2017; Layek et al., 2018; Peterson et al., 2019), as well as digital applications (Bachmann et al., 2015; Park, 2017; Verrucci et al., 2016), and the internet (Bachmann et al., 2015; Webb et al., 2010). Other ICTs that are proving to be relevant to all phases of DRM are the development of geographical information systems (GIS) (Hongbo et al., 2014) and remote sensing (Sausen & Lacruz, 2015; Troy et al., 2008). It is also interesting to consider the contributions of the Internet of Things applied to evacuation plans (Xu et al., 2018), early warning systems, and risk monitoring (Arshad et al., 2019).

Despite the clear potential of the ICTs, there is previous research pointing to the drawbacks and threats linked to their use. For starters, the role played by the predisposition of individuals in guaranteeing effective information transmission should be considered (Karanci et al., 2005). Furthermore, there is the danger of infiltration of harmful information in bidirectional systems (Giroux et al., 2013; Lindsay, 2011), which could be reduced by setting up an Information Management Team (Homier et al., 2018). What is more, the current world’s population does not benefit from a full mobile network and internet coverage, particularly when it comes to the most vulnerable population.

In spite of these shortcomings, the use of technology could enhance the speed of dissemination of information (Johnson, 1998) and mitigate the effects of disasters on the population (Palen et al., 2007; Shklovski et al., 2010). Moreover, the issue of electricity access and possible power outages during an active disaster scenario should also be taken into consideration. Possible solutions for this could lie in working offline and relying on automated backup options (Lindsay, 2011; UNDRR, 2019). An ever-expanding number of digital applications have been developed in this regard and require systematic reviews (Bachmann et al., 2015).

### The aim: evaluating the adequacy of mobile applications for reducing the adverse effects of natural hazards

Risks linked to natural hazards are subjected to change, uncertainty, and growing threats and hence require flexible solutions that can adapt on a real-time basis. At the same time, there is a proliferation of digital applications (apps) with great potential for reducing the adverse effects of natural hazards, in that they would improve communication at all stages of DRM (pre- and post-event). Yet, few studies have assessed this potential, all the more so if the parameters of risk exposure and vulnerability of each country are taken into consideration. In view of this, this research aims to assess the adequacy and viability of mobile apps worldwide for disaster risk reduction, linked to some of the current deadliest natural hazards, i.e. earthquakes, cyclones, and floods. Are existing disaster-related mobile apps used so far in the deadliest natural events adequate? Do they have the potential to be applied in high-risk countries?

## Materials and methods

To answer these questions a two-part methodology (Fig. 2) was developed. First off, an analysis of a random sample of the risk-related mobile apps available in high-risk countries was performed to extract conclusions about their adequacy, covering their characteristics and comparing them with the existing literature.

**Fig. 3:**
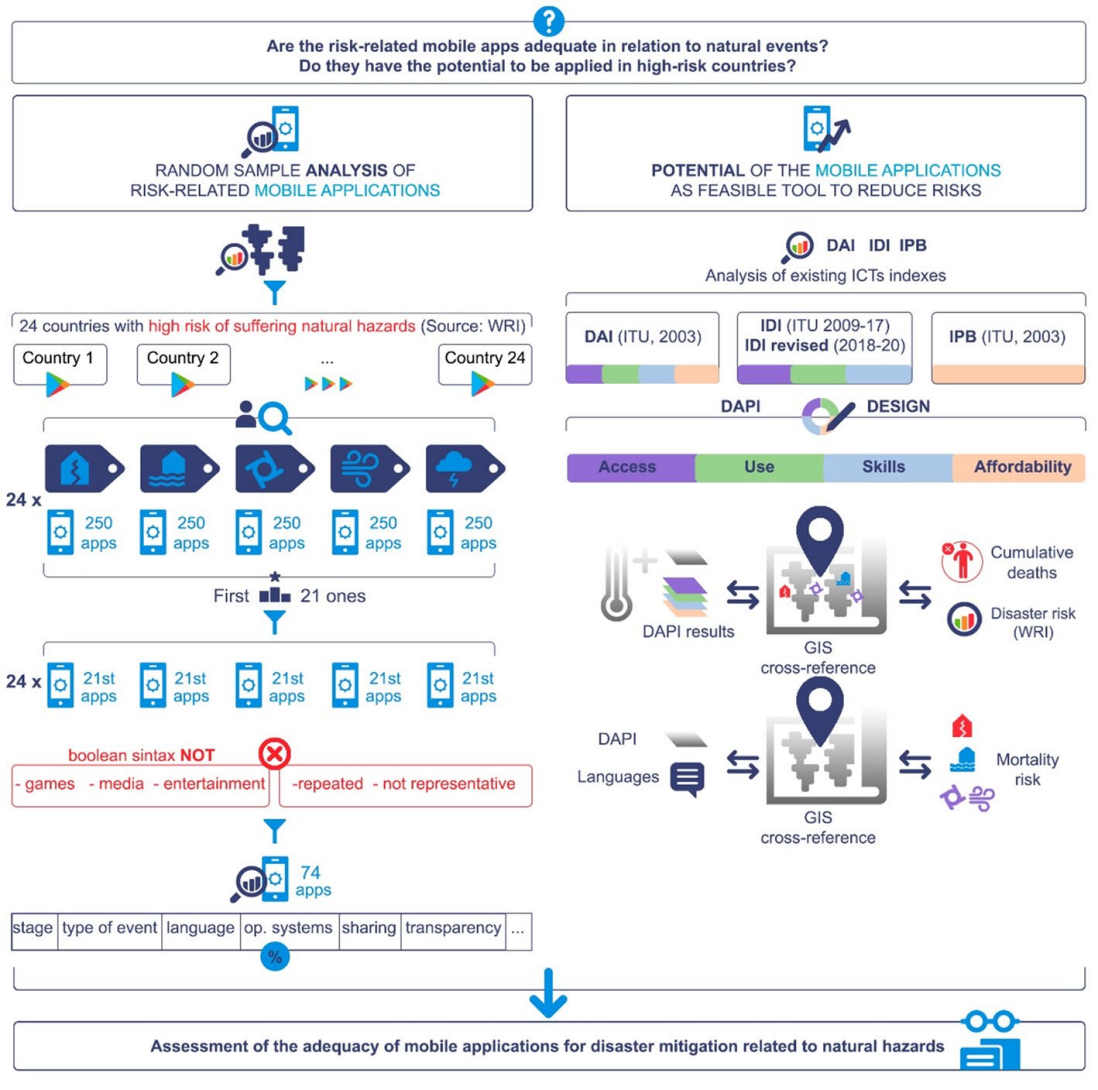
Research methodology flow

Moreover, an appraisal of the potential of mobile apps (according to their access, use, affordability, and skills) was conducted per country, using the indicator proposed by the authors: the DAPI (Digital Application Potential Index). The DAPI and each of its components were cross-referenced in GIS with the disaster risk of each country provided in the existing WorldRiskIndex (WRI), as well as with each selected hazard (hurricanes, earthquakes, and floods) and cumulative deaths by subregion. Each analysis is illustrated via visual maps to facilitate its understanding (Fig. 3).

### Random sample analysis of risk-related mobile applications available in high-risk countries

In order to make the research feasible, the scope of the sample for a preliminary analysis was limited to 24 countries across the world, based on their high risk of exposure according to the WRI 2019 (Mucke, 2019). The search was performed by region and country, mainly with the Android OS in mind, since 74.14% of smartphones currently use it as an operating system (Stats, 2019). In addition, the Google Play Store application was the main search engine, due to being available in most phones.

The Google Play Store does not arrange the applications associated with natural hazards in a single category (Google, 2020), and there is no global consensus among developers in terms of indexing and search tags, which also applies to natural hazard applications (Strzelecki, 2019). Hence, five keywords related to the deadliest natural hazards were determined: “Earthquake”, “Flood”, “Hurricane”, “Cyclone”, and “Storm”. The keywords were translated into each country's official languages, and the search was carried out in the Play Store of each respective country. Following this procedure, the Play Store displayed a maximum of 250 applications per keyword search and country, returning a first sample of 30,000 (250 × 5 × 24). The first twenty-one results per keyword were selected. Their order of appearance relies on a ranking system based on user ratings, downloads, and other internal criteria of the search engine (Karagkiozidou et al., 2019). The Google algorithm presents limitations related to the display order of the applications, which depends on geolocation, navigation preferences, and type of smartphone, to name a few.

Afterwards, an advanced search was performed using Boolean operators. The terms “-games”, “-media”, and “-entertainment” were applied to exclude off-topic games and apps, thus narrowing down the results and improving accuracy. Finally, unrepresentative or repeated apps were manually discarded, resulting in a final selection of 73 mobile apps.

The latter was classified into two DRM stages: pre-disaster and trans-post-disaster. To obtain an overview of these applications and evaluate them, a few useful parameters were collected, such as the compatibility with Android or IOS operating system, language options, and the possibility of sharing warning messages on social networks. The transparency and reliability of each application were assessed through the identification of the type of developer, the clarity of given and received information, and who has access to it. These aspects are relevant to ensure the confidential treatment of the data, prevent their alteration or access for advertising purposes, as well as an efficient coordination at the institutional level.

### Evaluation of the risk reduction potential of mobile applications

Over the years the International Telecommunication Union (ITU) has developed several indicators related to the potential of ICTs, most of them in reference to technology accessibility in general. Of particular interest to the research is the Digital Access Index (ITU, 2003), which measures the overall ability of individuals in a country to access and use ICTs. It is composed of five categories (infrastructure, affordability, knowledge, quality, and usage) and eight indicators. In 2009 they created the ICT Development Index (IDI) to assess and benchmark developments in ICTs. The original IDI was updated every year till 2017 (ITU, 2017). Compared to the Digital Access Index, the IDI redistributes quality in access and use categories, while discarding affordability (Table 2).

**Table 2:**
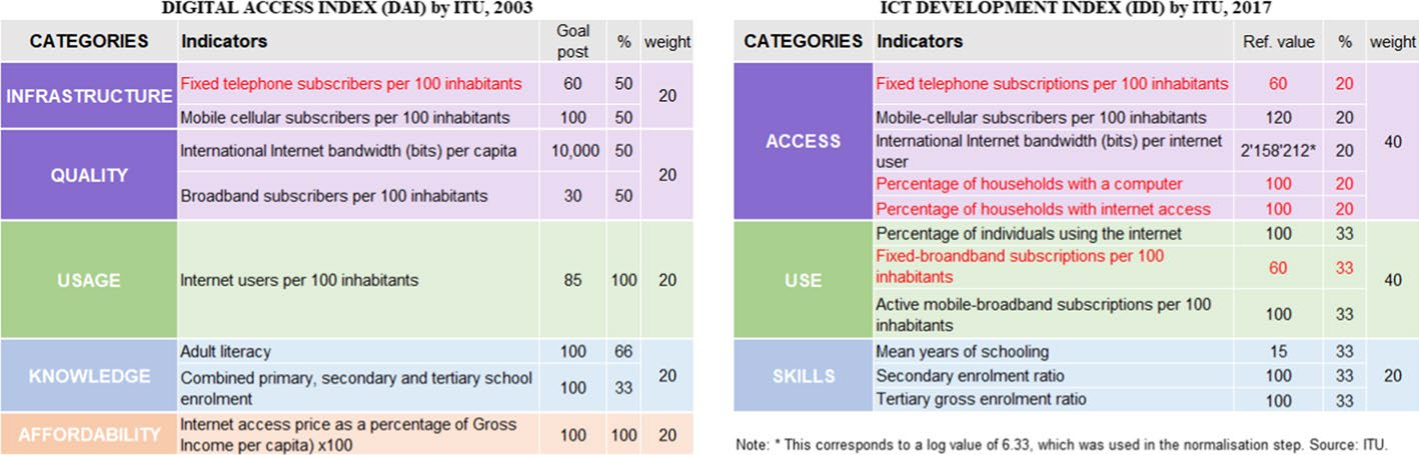
Comparative table of the Digital Access Index (ITU, 2003) and ICT Development Index by ITU (ITU, 2017)

The indicators and categories have been rearranged to facilitate the comparison among similar items

In response to technological developments, a revised ICT Development Index has been updated every year (ITU, 2019, 2018, 2020), adding more indicators. However, according to the ITU Secretariat, the revised IDI indicates that the methodology developed for the new indicators lacks robustness. For affordability, the ITU created a specific indicator: the ICT Price Basket 2019. This index measures ICTs’ relative price (fixed telephone, mobile cellular, and fixed broadband) as a percentage of gross national income per capita (GNI p.c.) (ICT et al., 2019).

On the basis of these previous indicators, the authors put forth a new composite indicator conforming to the purposes of the research, i.e. the Digital Application Potential Index (DAPI). The latter stems from the combination of the 2003 Digital Access Index, which includes affordability among its components, and the 2017 ICT Development Index. Affordability is a key parameter for evaluating the adequacy of mobile apps as a tool for risk management, as these must be accessible to the most vulnerable, and thus, the parameter of affordability was included with the most updated ICT Price Basket database. It must be pointed out that only the most accessible option for vulnerable users was used, i.e. the cheapest mobile service with data option (data and voice low consumption) from the indicators in the ICT Price Basket. Since the scope of the research only includes the evaluation of mobile apps, the fields indicated in red (Table 2) regarding computers, fixed telephone, and internet at home were excluded. Table 3 describes the final categories and indicators and all the source databases used for calculations in the development of the DAPI.^3^

**Table 3:**
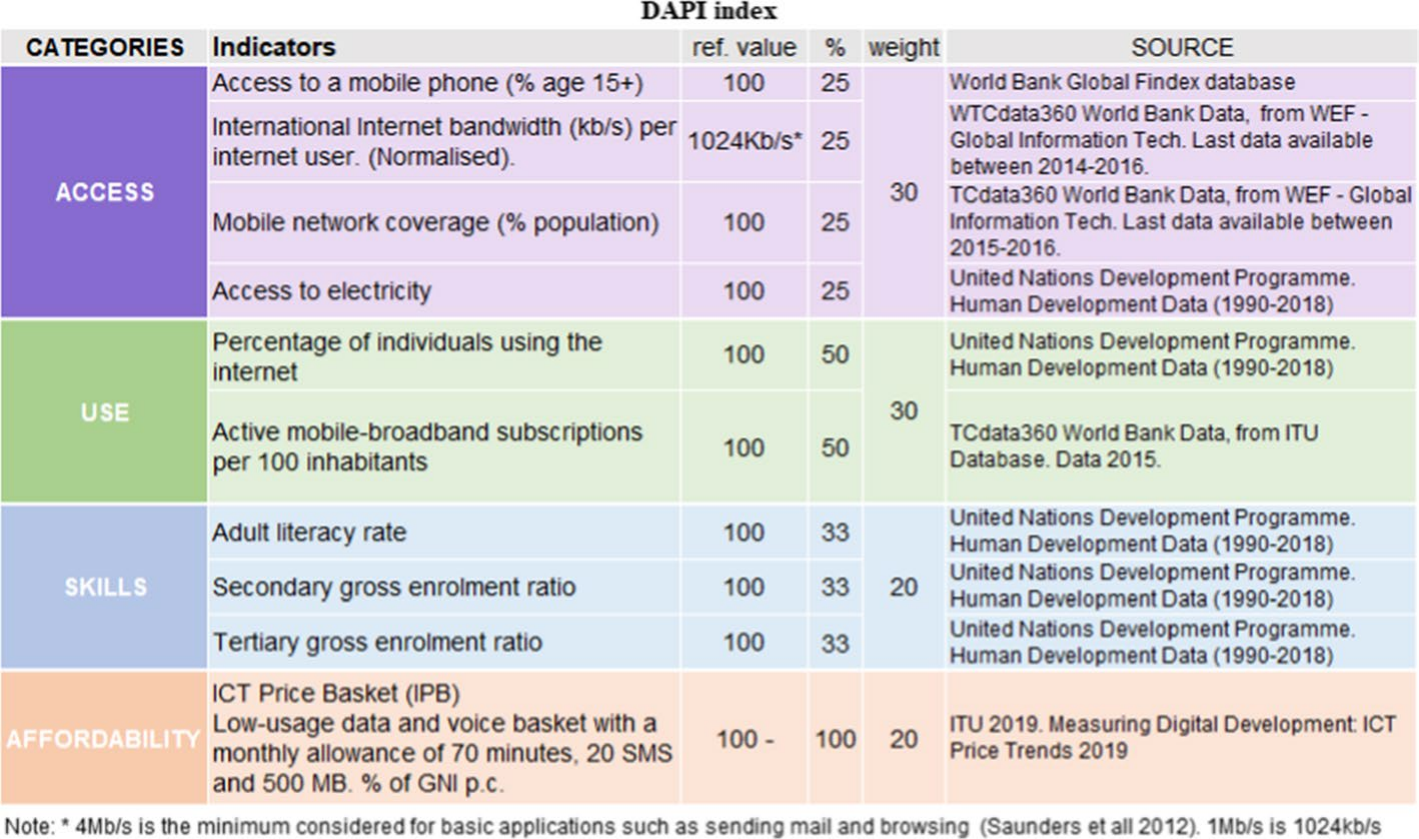
Description of the categories and indicators considered in the development of the Digital Application Potential index (DAPI).

To discuss the potential of digital apps in risk reduction in different countries, as well as the measures for rendering them usable, the DAPI and its components were cross-referenced in GIS with the disaster risk of each country listed in the existing WRI (Mucke, 2019). The WRI establishes the relationship between the exposure to natural events and the vulnerability of each country. All these values were added as fields in each country through GIS and displayed in maps. To facilitate the discussion of findings, the DAPI results were divided into quintiles, following a visual criterion according to which the darker the colour of the country in the map, the less viable will mobile apps be. In addition, for each component of the DAPI, the values were averaged, and the countries were grouped by risk category. The maximum and minimum values were included in a tabulated form to discuss the range of variability.

## Results and discussion

### General overview of mobile apps

Nowadays, mobile apps have become an additional tool of communication on top of traditional ones, such as television or radio (Lindsay, 2011; Wahyu et al., 2012). However, it must be highlighted that this type of apps should not depend exclusively on mobile data, so as to guarantee the highest percentage of population coverage inclusive of users that lack mobile data access or in the event of facing network failure. Social networks (Hong et al., 2017; Peterson et al., 2019) and SMS (Cioca et al., 2009; Webb et al., 2010) are among the most common alternatives to mobile apps. These media have proven to be beneficial not only for mitigation purposes and accessing a large number of the population, but also to re-establish communication between people and within the community (Shklovski et al., 2010), to create collaborative maps (Doktor & Giroux, 2011; Dunn Cavelty & Giroux, 2011; Layek et al., 2018), to extend the management capacity of safeguard services, and to prioritise actions that could have been disregarded by public services (Giroux et al., 2013; Norris, 2017; Palen et al., 2007; Wang et al., 2016). Nonetheless, only 28% and 36% of the applications assessed in this research permitted information sharing through other social networks or SMS in both phases: pre- and post-trans disaster, respectively, which is significantly low. Moreover, globally only 30% allowed information (photographs, videos, and reports) to be shared, which can be very useful for determining the extent of damage to the territory. The addition of other features in information sharing, such as collaborative maps, would be of great interest.

Another striking finding was the importance of interconnecting all phases and disaster types to improve the effectiveness of the humanitarian response (Park, 2017) and ergo offer advice and warnings tailored to each area, along with the scale and type of disaster. Notwithstanding this, only 10% of digital applications analysed presently cover both pre- and trans-post-disaster phases (7 apps), in contrast to the 77% focussing on the pre-disaster phase and the 14% centred on the trans-post-phase. Additionally, the pre-disaster approach seemed rather insufficient, solely depending on warnings and advice on how to proceed against risks. On the other hand, the trans-post-disaster apps, included those intended to collect information, mostly focussed on locating citizens (75%).

Furthermore, most mobile apps limit their coverage to a single type of natural event (78% of the sample), so multi-hazard applications should be promoted (Verrucci et al., 2016) for resource optimisation and efficient stakeholder coordination.

What is more, public entities could provide information about public facilities that cover basic needs, such as water and food, healthcare facilities, and shelter locations, apart from providing safeguard emergency services (Park, 2017). From this point of view, the use of a single application would optimise and accelerate response management (Park, 2017; Verrucci et al., 2016) and could strengthen local capacities among regions for disaster preparedness. Moreover, using artificial neural networks (Pashazadeh & Javan, 2020) and artificial intelligence (Abarca-Alvarez et al., 2019) for building forecasting models (Ogania et al., 2019; Williams & Lück-Vogel, 2020) could have a beneficial impact, and it could be adequately complemented by spatial–temporal detection systems (Yu et al., 2020) and the Internet of Things (Arshad et al., 2019; Xu et al., 2018).

In addition, a team of specially trained professionals must be responsible for the DRM communication to secure its effectiveness (Homier et al., 2018), as happens with the Red Cross and the Surge Information Management Support. This team is of great importance to empathically transmit collective recommendations and specific messages at a time of social pressure (Giroux et al., 2013; Grassau et al., 2019; Hong et al., 2017). Per contra, the latter can exercise control over the news delivered as well as over users with malicious intent (Giroux et al., 2013). According to previous research, this specialised training should be extended to local leaders to ensure the effectiveness of the preparedness actions (Verrucci et al., 2016).

However, the strategy laid out above clashes with the fact that 55% and 79% of the pre- and trans-post-disaster applications, respectively, were found to be developed by private companies. The high rate of app development in the private sector stems from strategic and market exploration agreements, perceiving social issues to be new investment opportunities. More often than not, the information collected may be used for statistical and marketing purposes without the users' knowledge or consent (Kemper & Kolkman, 2019). Due to weak data protection and privacy legislation, the "datification" in medium- and low-income countries with high levels of risk and vulnerability could lead to unwanted scenarios of information monopolisation by the private sector (Roth & Luczak-Roesch, 2020). To prevent the misuse or abuse of citizen information collected by third parties, government agencies and academic institutions created protocols such as Open Data, FAIR data (Findable, Accessible, Interoperable, Reusable) (Wilkinson et al., 2016), and FACT data (Fairness, Accuracy, Confidentiality, and Transparency) (Van der Aalst et al., 2017). These promote the need for policies that endorse reliability, transparency, citizen empowerment and control over their data, in a way that does not hinder private development, preserves citizens’ rights, and assures availability for researchers and organisations. A strong public service can protect the population from private interest while enforcing information reliability together with leading management transparency.

### Evaluation of the adequacy of the mobile applications

From a general point of view, a trend was identified in which the lower the disaster risk, the higher the potential of using mobile apps (DAPI value) (Table 4). This statement is true when considering the minimum DAPI values, yet some discrepancies were observed in regard to the maximum DAPI values for low and high disaster risk categories, as well as regarding mean DAPI values. This is due to the extremely wide range of values among countries within the same risk category. This evidences that, to date, mobile apps cannot be used as the sole communication tool in countries with high levels of disaster risk.

**Table 4:**
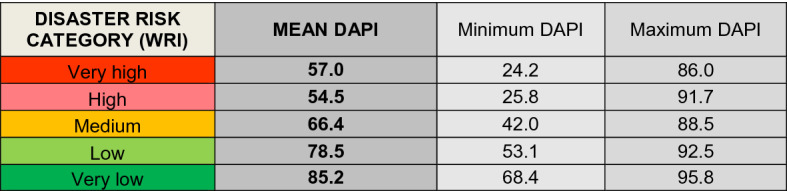
DAPI results by country group, according to the WRI from the World Risk Report 2019 (Mucke, 2019)

Mean, minimum, and maximum values

#### Access component of the DAPI and risk

**Fig. 4:**
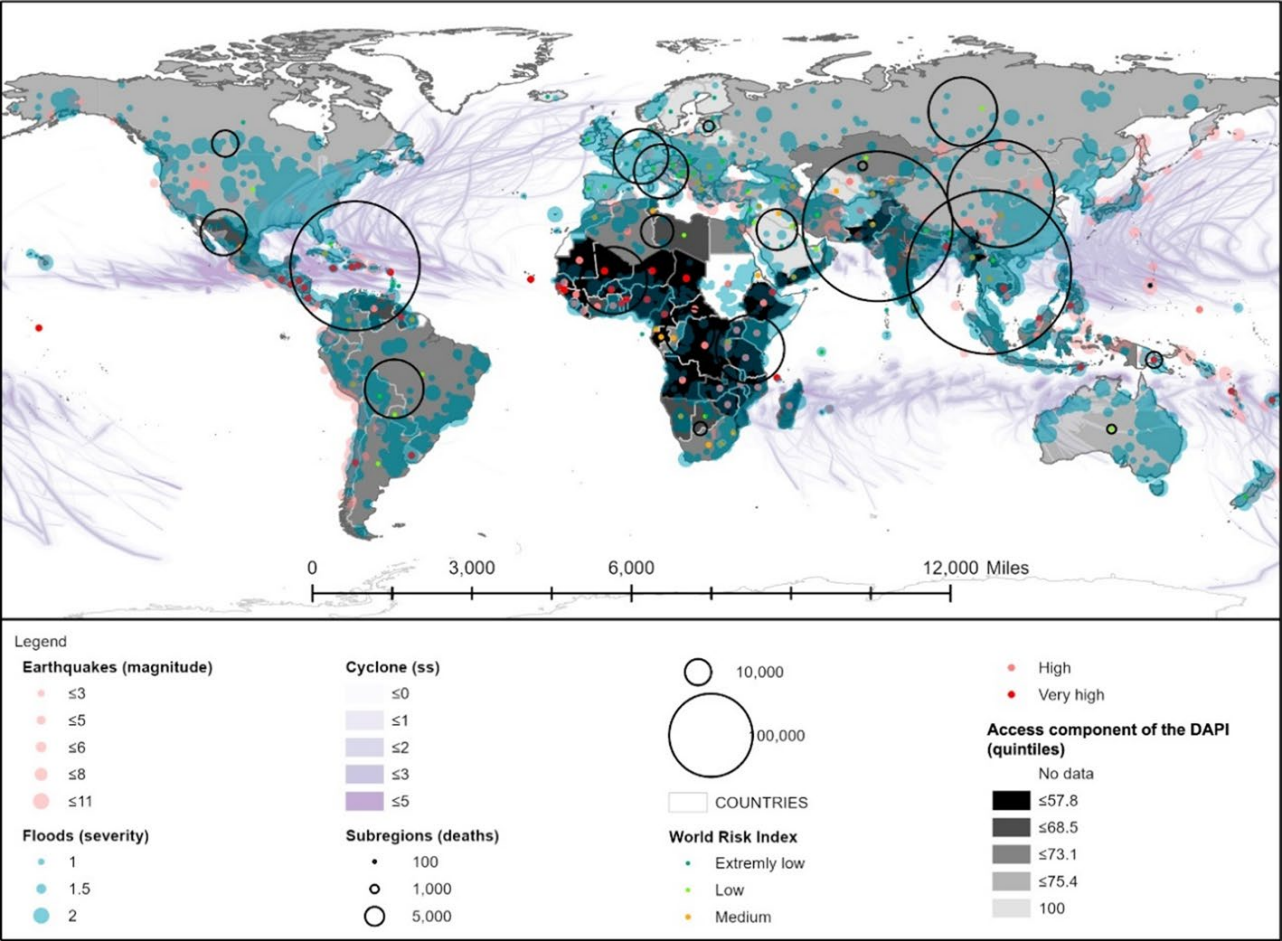
Access component of the DAPI related to the WorldRiskIndex, natural events, and deaths by subregions. Subregions, flood events, major earthquakes, and cyclones as in Fig. 3. Shapefile countries: Esri, Garmin, CIA World Factbook (*References: Regional and World Maps—The World Factbook—Central Intelligence Agency*, 2020). Access component of the DAPI as explained in the methodology. WorldRiskIndex (WRI) from World Risk Report 2019 (Mucke, 2019), linked to countries by ISO code

Four indicators were considered for the evaluation of the access component: mobile phone access, expressed as the percentage of population over 15 years old; international internet bandwidth (kb/s) per internet user (normalised); mobile network coverage (expressed as the percentage of population); and electricity access. The WRI-related access component of the DAPI can be observed in Fig. 4.

It must be stressed that the countries with the lowest DAPI access are located in Africa, together with Haiti, which occupied the 14th position with a value of 44, and Pakistan (25th position with 52). In the case of Haiti, the low access index is due to the low bandwidth, although the rate of mobile phone access is 68% and the network coverage is 63%. Hence, in this case, an improvement of the bandwidth and electricity would be a priority since only 45% of the population can benefit from it.

The subregions of South and Southeast Asia and the Caribbean stand out for their significant number of deaths. These areas display a DAPI access average of 66%, 67%, and 64%, respectively. Nonetheless, South and Southeast Asia scored high rates of access to mobiles, electricity, and network coverage compared to the Caribbean, with the exception of Pakistan, which presented an access rate to mobile phones of 54% and electricity of 71%. Again, the authors highlight that country, region, and subregion-specific analyses must be conducted since, for instance, within the Caribbean subregion, the Dominican Republic catches the eye for its high rates of access, i.e. 81%, 99%, and 100% of access to mobile phones, network coverage, and electricity access, respectively. Table 5 displays the correlation between the disaster risk classification according to the WRI and the mean DAPI access.

**Table 5:**
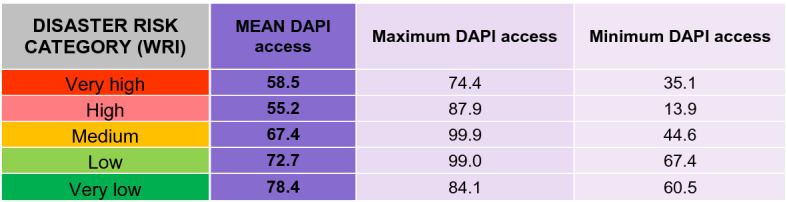
DAPI access results by country group, according to the WRI from the World Risk Report 2019 (Mucke, 2019)

Mean, minimum, and maximum values

Even though the lowest disaster risk was found to often be linked to the highest technology development, this is not always the case, as happens with the maximum DAPI access value for very low disaster risk categories, as well as the minimum DAPI access values.

Thus, the relationship between said variables and the best course of action will be area-specific. Given that the most vulnerable people have no access to electricity or internet connection, any strategy that is implemented must benefit large isolated areas and the community as a whole. The analysis suggested that the use of digital applications is insufficient to ensure a timely response to natural hazards. Other alternatives such as television, radio, and community leaders education must be considered (Lindsay, 2011; Wahyu et al., 2012).

##### Usage component of the DAPI and risk

The usage of mobile phones and internet is one of the key parameters in the proposal for mitigating risk disasters. As aforementioned, this component is based on the percentage of individuals using the internet as well as the active mobile broadband subscriptions per 100 inhabitants.

**Fig. 5:**
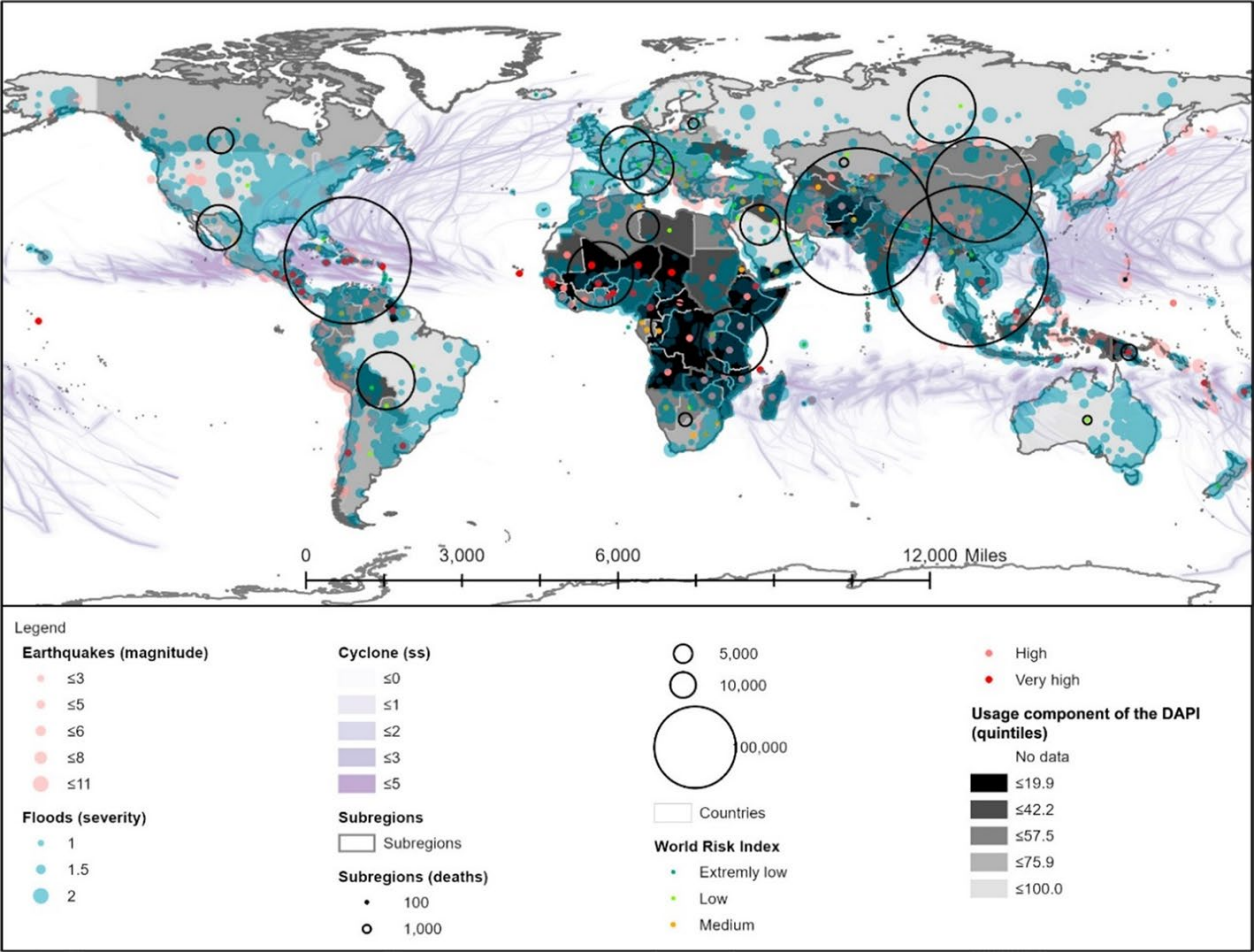
Usage component of the DAPI (average of percentage of internet users and active mobile broadband subscribers per 100 inhabitants). Subregions, flood events, major earthquakes, and cyclones as in Fig. 3. Shapefile countries as in Fig. 4. Usage component of the DAPI as explained in the methodology. WRI as in Fig. 4

The results are plotted in Fig. 5, in which the darkest colour indicates the lowest value of the component. Africa stands out due to owning the lowest usage rate, followed by countries in the Caribbean, Central America, and Asia, such as Haiti (16.3%), Nicaragua (17.5%), Tajikistan (17%), Lao (19.9%), and Nepal (21%).

The last two regions are of great interest for their extremely high number of deaths. Among them, the lowest usage of technology was found in South Asia, Polynesia, Central Asia, and Central America, with an average DAPI usage component of 29.7%, 35.4%, 36.5%, and 41.6%, respectively. On the opposite end, there is the Caribbean, where the average usage is 51.1%, albeit with a broad spread of usage data values between countries, ranging from 16% for Haiti to 75.9% for Saint Kitts and Nevis, and 57.2% for the Dominican Republic.

Once more, when the disaster risk category is analysed together with the DAPI usage component (Table 6), there is no clear correlation regarding maximum and minimum values. The considerable differences between the riskiest countries compared to the ones with low risk are noteworthy. The use of technology is 41% lower in countries with a very high risk of disaster compared to the very low-risk ones. Yet, this cannot be explained by differences in accessibility, as these groups diverged only slightly in this regard, and thus, it can be stated that the use of technology is not linked to its access.

**Table 6:**
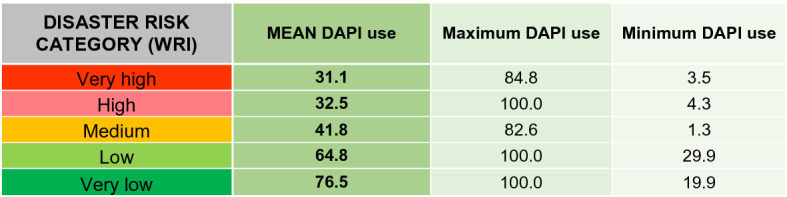
DAPI usage results by country group, according to the WRI from the World Risk Report 2019 (Mucke, 2019)

Mean, minimum, and maximum values

##### Skills component of the DAPI and risk

The educational level of the population is another parameter that impacts its degree of understanding and subsequent capability of following advice, as well as the level of engagement between the emergency services and the population. In fact, the population may need training for following advice and reporting data (Homier et al., 2018). In this sense, the digital skill component indicates the population’s familiarity degree with this type of technology, in that it evaluates the adult literacy rate (ITU, 2019), i.e. the capacity to read information, as well as the second and tertiary gross enrolment ratios, i.e. the number of students in relation to the number of people in school age, indicating their level of participation, and their possible role in an emergency situation. In this case, the lack of content in local languages could present a barrier that should also be taken into account (ITU, 2019).

**Fig. 6:**
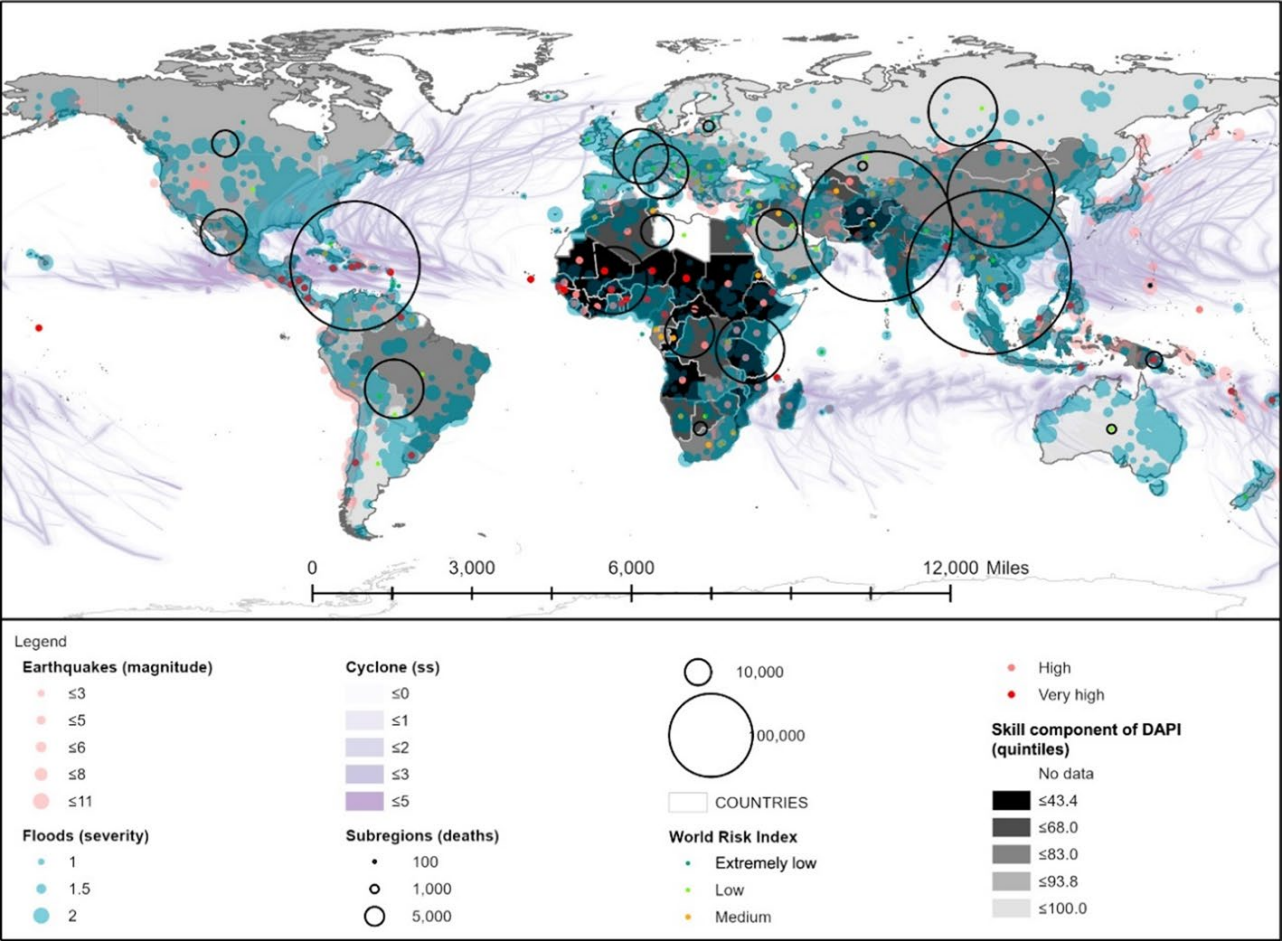
Skill component of the DAPI (average of literacy rate and secondary and tertiary enrolment). Subregions, flood events, major earthquakes, and cyclones as in Fig. 3. Shapefile countries as in Fig. 4. Skill component of DAPI as explained in the methodology. WRI as in Fig. 4

Figure 6 shows that the lowest values of the DAPI skills component were achieved in Africa (Sub-Saharan region) and some countries of South Asia, such as Afghanistan (32.2%) and Pakistan (37.5%), with a subregional average of 54%. Concerning the number of deaths, South Asia is the worst region among those with low skill rates. By contrast, the high skill rates in the Caribbean subregion can be highlighted, averaging 83.4%, with a literacy rate of 97%, a secondary enrolment rate of 95%, and tertiary enrolment dropping to 48%. This is shortly followed by Central America, with one of the lowest averaging rates, i.e. 67%, and a tertiary enrolment of 33.9%. The latter is akin to the rate achieved by Western Asia, achieving 74.2% on the skill component of the DAPI.

According to the WRI, the highest risk countries exhibited high rates of literacy of about 86.4%, which decreased to an average of 75% for the secondary enrolment. In the case of tertiary enrolment, however, values plunged to 25.9%. The worst values among the top ten countries were found for Guatemala, Bangladesh, and Papua New Guinea, with a DAPI skills component of 52%, 52.6%, and 55.2%, respectively.

The correlation between the disaster risk category and the skills component of the DAPI can be observed in Table 7: The countries with a very high risk showed the lowest maximum and minimum skills category of the DAPI compared to the remaining ones. In spite of this, anew the very high and high disaster risk categories exhibited a substantial data range that narrows down as the risk of the category decreases, and therefore recommendations should be case-specific.

**Table 7:**
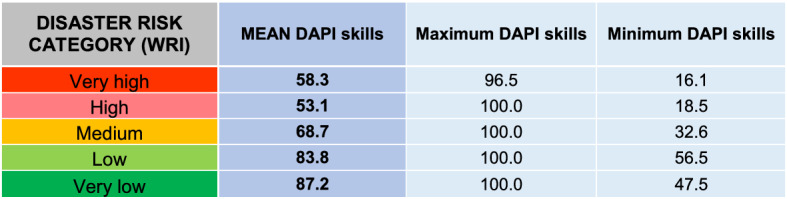
DAPI skills results by country group, according to the WRI from the World Risk Report 2019 (Mucke, 2019)

Mean, minimum, and maximum values

##### Affordability component of the DAPI and risk

**Fig. 7:**
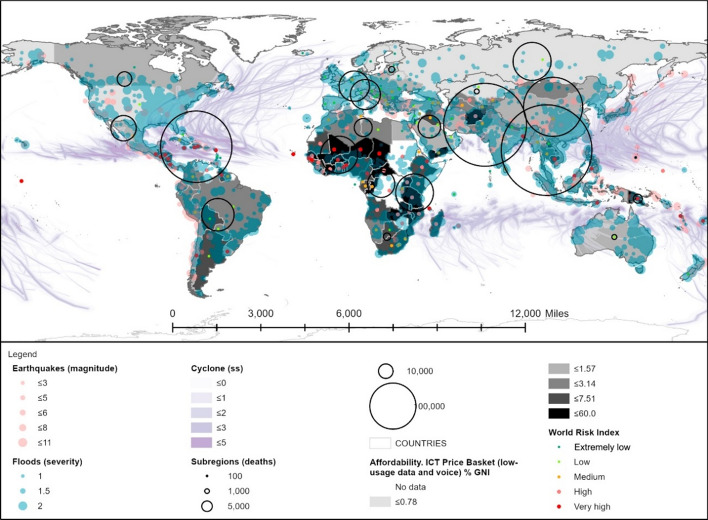
Affordability component of the DAPI (mobile data and voice low-usage basket) related to the WorldRiskIndex, natural events, and deaths by subregion. Subregions, flood events, major earthquakes, and cyclones as in Fig. 3. Shapefile countries as in Fig. 4. Access to electricity: percentage of the population with access to electricity. Affordability of mobile data and voice low-usage basket, as a percentage of GNI per capita. WRI as in Fig. 4

The main limitation associated with the use of mobile apps lies in the technology cost (ICT et al., 2019). The cost of the low data and voice basket consumption expressed relative to the GNI p.c. strongly differs between developed countries (1% of the GNI p.c.), developing countries (7.5% of the GNI p.c.), and least developed countries (17%). In addition, in the latter two cases, the price difference with respect to low and high data and voice basket consumption is noteworthy, with low data and voice basket consumption being very prevalent in low-income countries (ICT et al., 2019). For this reason, this category was considered as a reference for the affordability component of the DAPI (Table 3). A price reduction would accelerate the increase in mobile phone penetration (ICT et al., 2019), albeit not being the only key aspect. Additional ones, such as the educational level and the use of local languages, should also be taken into account (ICT et al., 2019). Figure 7 illustrates the inverse correlation between the highest DAPI values and the lowest consumer affordability, thus revealing a low potential for introducing mobile apps in those areas.

The lowest values related to the affordability component of the DAPI were found in South America, with an average of 2.9. For its part, the regions with the highest death rate, i.e. Southeast Asia, South Asia, and the Caribbean, exhibited an average of 2.6, 3.0, and, 4.4, respectively. Nonetheless, there are high discrepancies within them, in particular regarding Haiti with 13.8 and Barbados with 1.6 in the case of the Caribbean, and Afghanistan with 13.5 compared to the 0.44 for Sri Lanka in South Asia.

Furthermore, when the average consumption is contrasted with the WRI disaster risk category (Table 8), it can be observed that only countries with low and very low risk of disasters related to natural hazards maintain affordable prices below 2% of the GNI p.c. In what high and very high-risk countries are concerned, the mean cost amounts to 11.3% and nearly 12% of the GNI p.c. (Table 8) with peaks in African countries, such as Liberia (60%), Niger (56.8%), and Central African Republic (48.1%). Among the highest risk countries, Papua New Guinea (22.2%), Central America (Guatemala 10.9%), Honduras (10.4%), Nicaragua 9%), as well as Haiti (13.8%) and Afghanistan (13.5%) stand out. Southeast Asia is an area of particular interest for its affordability values, with values of 2.5%, while pertaining to a high-risk category.

**Table 8:**
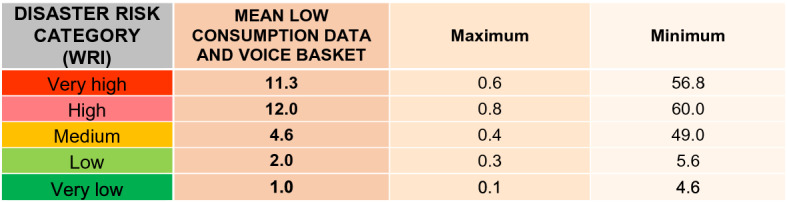
Results by country group, according to the WRI from the World Risk Report 2019 (Mucke, 2019)

Mean, minimum, and maximum values. Consumption expressed as a percentage of GNI per capita. Created by the authors, adapted from ITU, 2019 and WRI 2019

#### Evaluation of each type of natural risk and the DAPI

Complementarily to the previous analysis, the DAPI was evaluated in relation to the type of natural events. Earthquakes, cyclones, and floods were considered separately.

##### Earthquakes and the DAPI

**Fig. 8:**
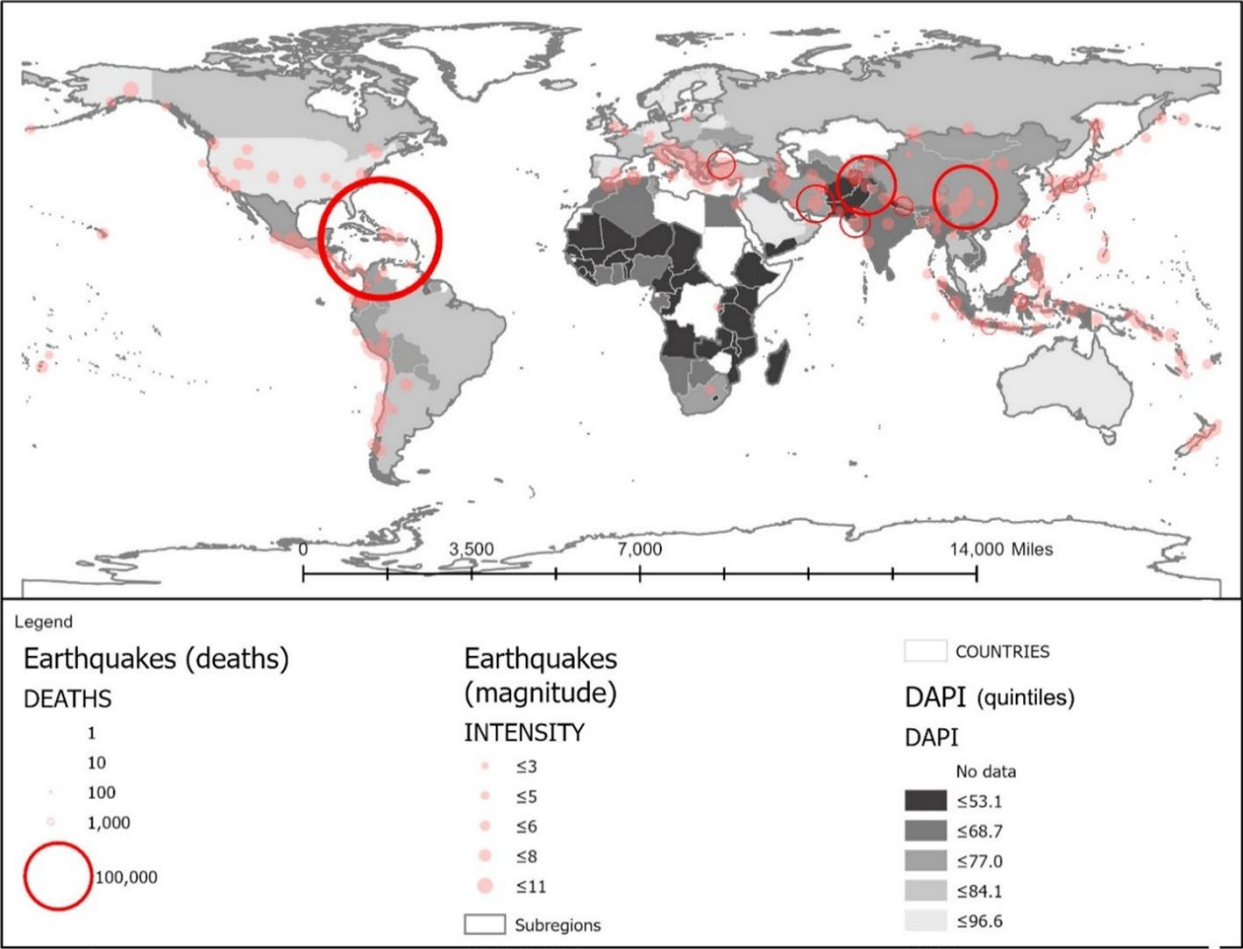
Correlation between earthquakes and the DAPI in each country. Subregions, major earthquakes by deaths, and severity as in Fig. 3. Shapefile countries as in Fig. 4. DAPI as explained in the methodology

In Fig. 8, the earthquake magnitude was overlaid with the DAPI. In the Caribbean area, the DAPI exhibited high values and high death rates linked to this type of natural event. Hence, technology should be considered as a strategy tool for risk disaster mitigation. The same applies to China, whose DAPI value stands at a medium range.

However, this strategy would not be feasible for some areas, such as Pakistan and Afghanistan, for which the number of mobile subscriptions (per 100 people) is 73% and 59%, respectively, but the number of internet users drops to 16% and 14%, respectively (*World Bank Open Data|Data*, 2020). This is also true for other areas, such as Syria, where mobile subscriptions amount to 13.61 million, i.e. 74% of its population, yet the number of internet users reduces to only 33% of the country's population (*World Bank Open Data|Data*, 2020).

This evaluation demonstrated anew that area-specific analyses are required. For instance, in some earthquake-prone countries such as Singapore, the number of mobile subscriptions is 8.37 million, which represents 144% of the population, and internet users are high, with 84% penetration (*World Bank Open Data|Data*, 2020). Therefore, in some countries, the appropriate measures will heavily rely on traditional media resources, such as television and radio advertisements, rather than mobile phones.

##### Hurricane and cyclone risk and the DAPI

**Fig. 9:**
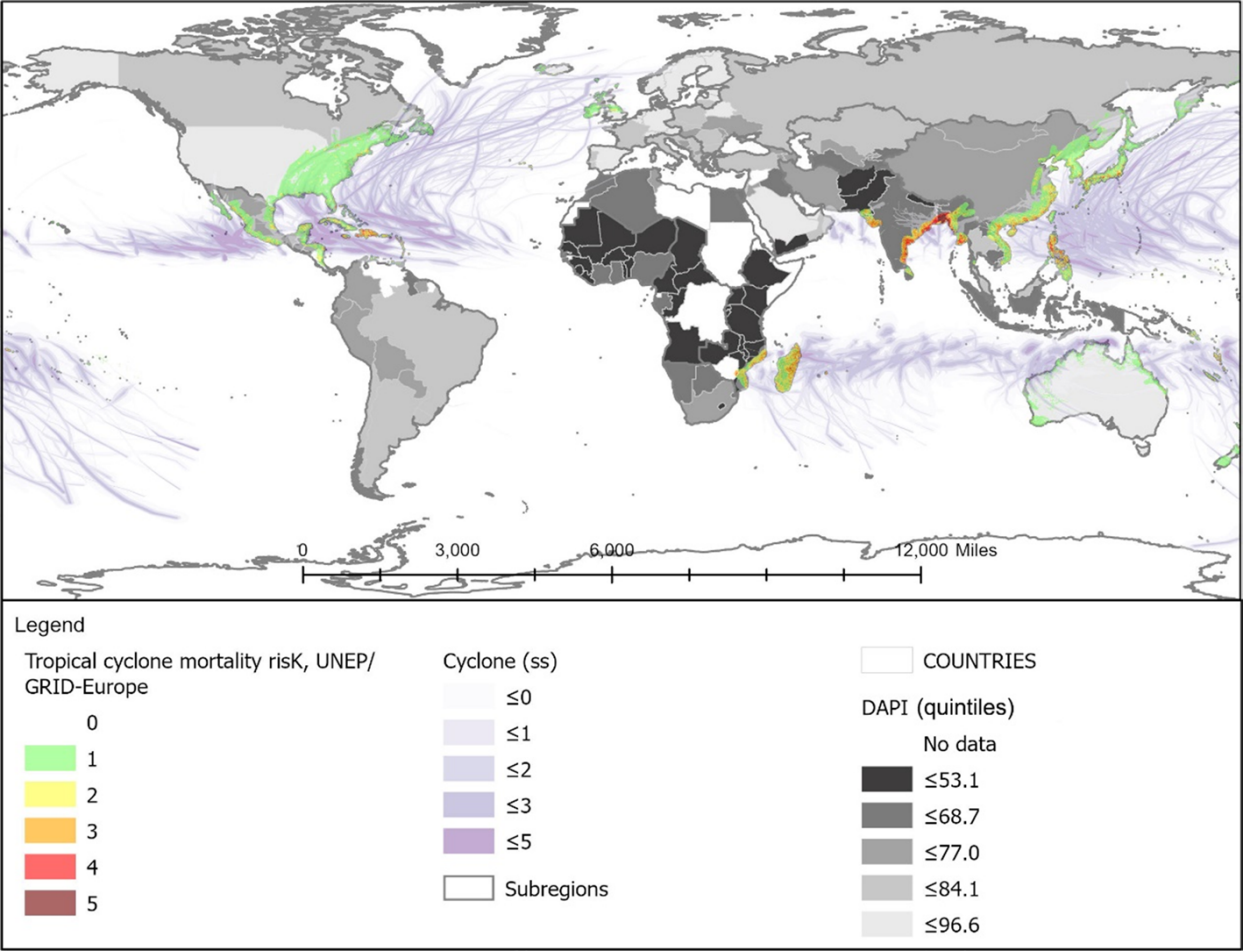
Correlation between cyclone risk and the DAPI in each country. Subregions, cyclones by category as in Fig. 3. Shapefile countries as in Fig. 4. Tropical cyclone mortality risk by UNEP/GRID-Europe (Global Risk Data Platform, 2020). Estimation of the global risk induced by tropical cyclone hazard. The scale of the estimated risk index ranges from 1 (low) to 5 (extreme). This product was designed by UNEP/GRID-Europe for the Global Assessment Report on Risk Reduction (GAR). DAPI as explained in the methodology.

Concerning hurricanes and cyclones (Fig. 9), a correlation was found between the DAPI in very high or high-scoring countries and the risk of natural hazards. In general, mobile apps can be considered an interesting tool, among others. The high expense brought about by internet connection must be taken into account while devising the implementation for mobile apps. In fact, in some countries, such as Haiti, while mobile subscriptions account for 61% of the population (6.85 million), internet users are reduced to 19%, with 2.1 million (*World Bank Open Data|Data*, 2020).

Mozambique and Madagascar are the exceptions to the rule, as being a high-risk country does not seem to relate to DAPI high values, therefore excluding the possibility of technology use.

##### Flooding risk and the DAPI

**Fig. 10:**
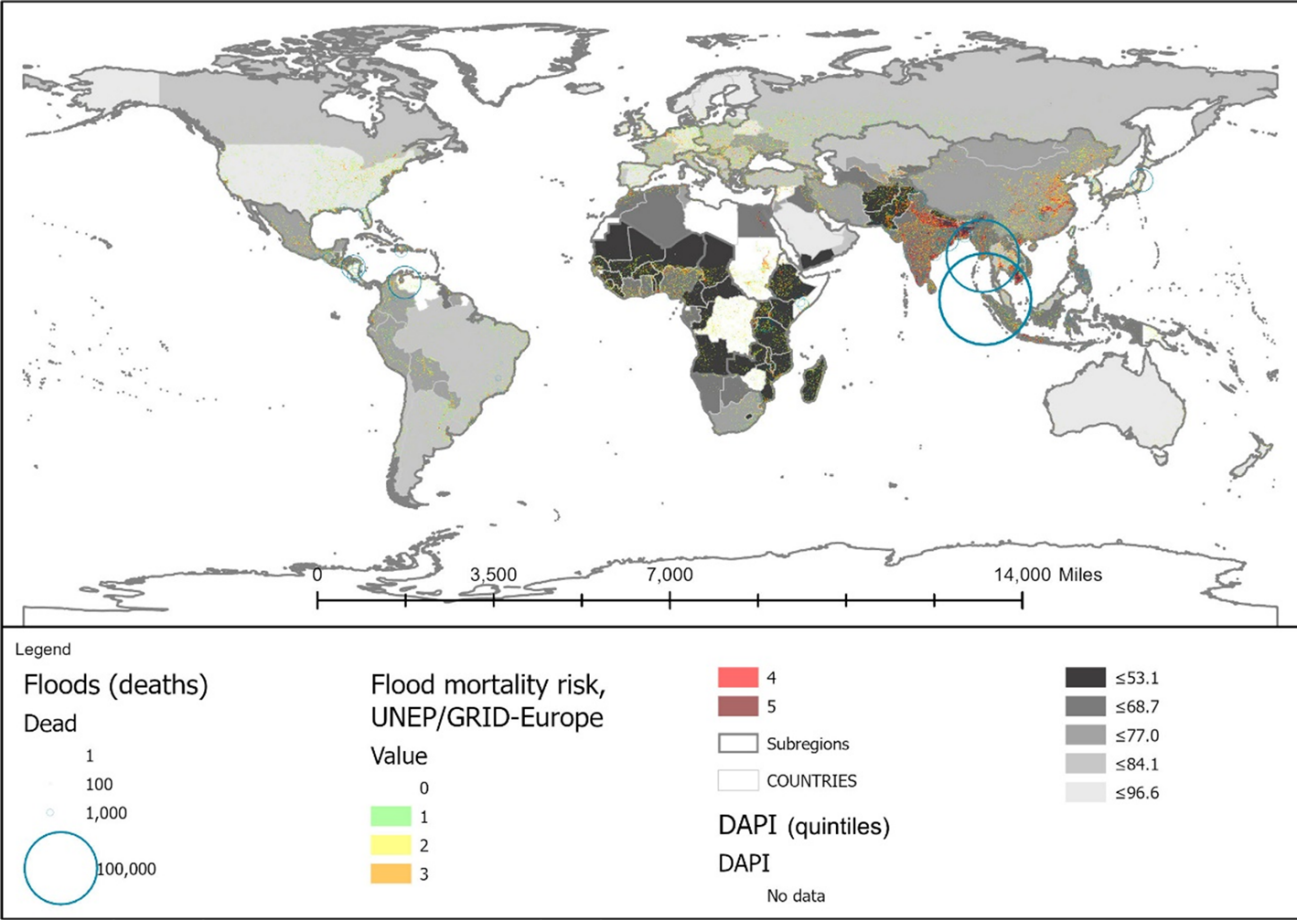
Correlation between flooding risk and the DAPI in each country. Subregions, floods by severity and deaths as in Fig. 3. Shapefile countries as in Fig. 4. Flood risk by UNEP/GRID-Europe. Estimation of the global risk induced by flooding. The scale of the estimated risk index ranges from 1 (low) to 5 (extreme). Designed by UNEP/GRID-Europe for the Global Assessment Report on Risk Reduction (GAR). DAPI as explained in the methodology

India, Nepal, Bangladesh, Cambodia, and some areas of China are the most vulnerable to floods (Fig. 10), and their DAPI was 57, 53, 54, 60, and 74, respectively. The affordability indicator for Bangladesh and India is noteworthy, at 1.4 and 0.9, respectively, with a price reduction strategy being a possible option for both of these cases. Their affordability data correlated with their usage of mobile technology, with 14.25% in Bangladesh and 22% in India, similar to the 21% found for Nepal, in spite of scoring higher (4.6) on the ICT Price Basket (ICT et al., 2019). The remaining parameters exhibited quite similar values.

Apropos of the death rate, the high-flooding risk of Myanmar is striking. The mobile phone coverage reaches 114% of its population, which falls to 31% when it comes to internet users. In like manner, Laos has a mobile subscription of 81% of the population, compared to 39% of internet users, i.e. 2.7 million people.

#### Other aspects to take into account

**Fig. 11:**
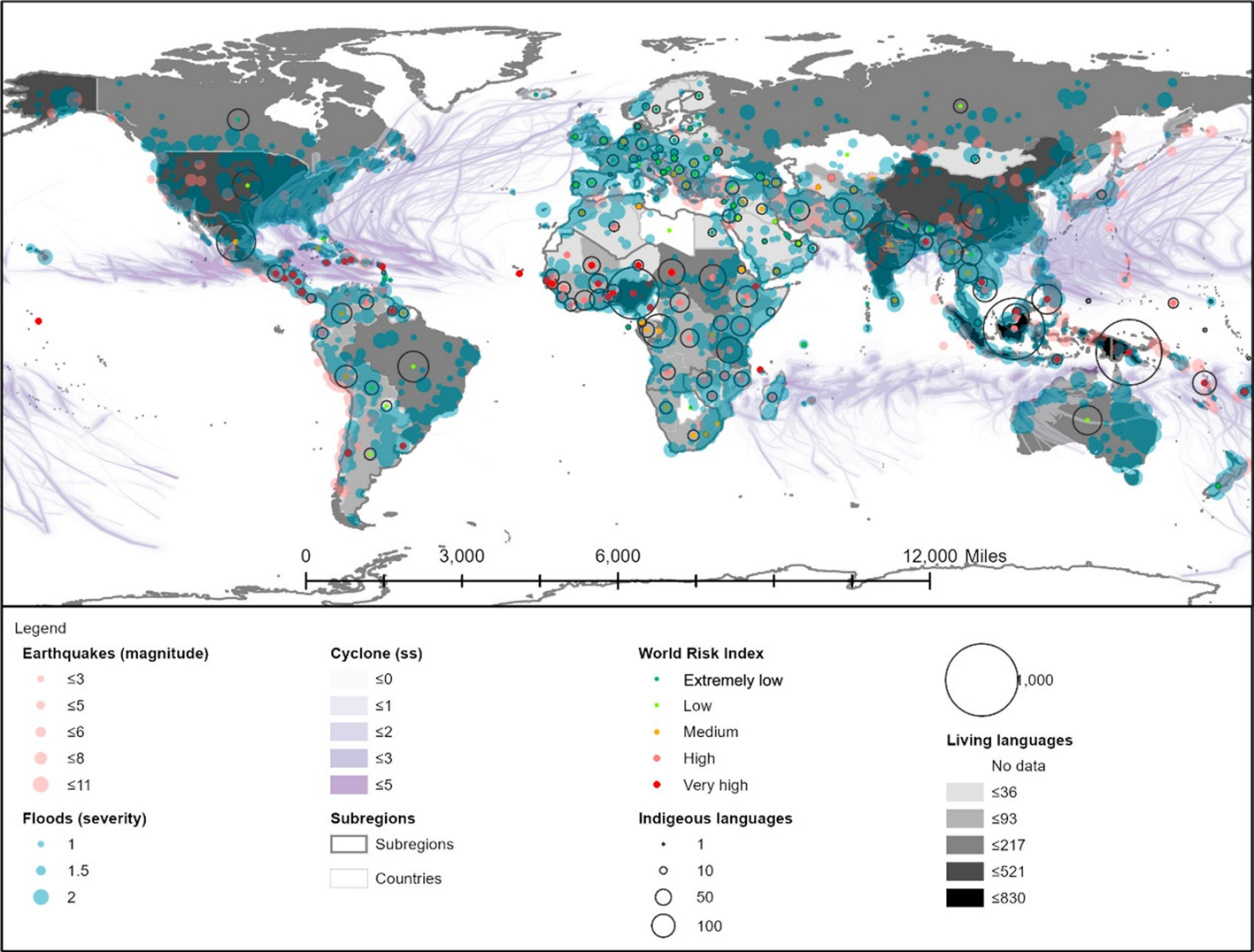
Languages and risk. Subregions, flood events, major earthquakes, and cyclones as in Fig. 3. Shapefile countries as in Fig. 4. WRI: as in Fig. 4. Living and indigenous languages (*Ethnologue: Languages of the World*, 2020; *UNESCO Atlas of the World’s Languages in Danger*, 2020)

A limitation that should be taken into consideration when aiming to reach the largest number of people possible is the language barrier (Fig. 11). English is the most widely spoken language (1,500 million) (*Statista Database*, 2020) and the most commonly used on the internet (*Internet World Stats—Usage and Population Statistics*, 2020), yet it is the native language of only 5.3% of the population (*Worlddata: The World in Numbers*, 2020). This excludes the population with a lesser degree of academic training, which usually matches the most vulnerable to natural hazards and, therefore, those most in need of risk-mitigating apps. In 2016, the number of registered illiterates achieved 13.75% of the population (*Our World in Data*, 2020). From this point of view, the use of local languages has been identified as an additional aspect that impacts the effective use of the internet (ITU, 2019) and, by extension, that of mobile apps.

Figure 11 illustrates the correlation between natural events and the use of indigenous languages. According to it, Papua New Guinea and Indonesia hold the highest amount of living languages, and henceforth, the use of dialects and different languages must be considered in the development of apps. The adaptation to local languages would also be required for scarce linguistic-variability areas, such as the Caribbean region.

Along the same line, Table 9 highlights that the highest linguistic variety takes place in the areas with a very high and high disaster risk. This entails that any efficient tool must be adapted to local and indigenous languages to reach the highest possible percentage of population.

**Table 9:**
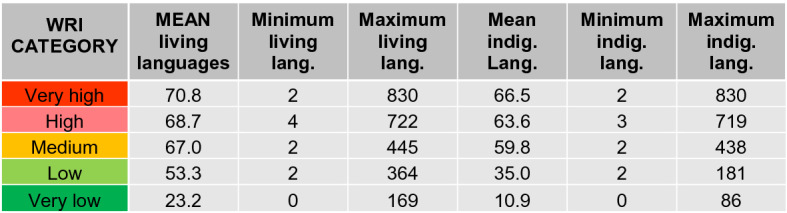
Languages by country group, according to the WorldRiskIndex from the World Risk Report 2019 (Mucke, 2019)

Living and indigenous languages based on Ethnologue (*Ethnologue: Languages of the World*, 2020) and UNESCO (*UNESCO Atlas of the World’s Languages in Danger*, 2020)

Despite the relevance of languages, the download platforms of apps do not provide information on the dialects in which these can be used. It is an important parameter to take into account for app development. At the same time, apps must be developed with a simple and intuitive graphical user interface, adapted to people with reading difficulties, and facilitate communication by audio for blind and illiterate people.

## Conclusions

The preliminary evaluation of the types of mobile apps evidenced that most of the apps limit their coverage range to only one stage of Disaster Risk Management (DRM) and one type of hazard event. In general, pre-disaster apps are based on warnings and advice on how to proceed against risks, while trans-post-disaster ones mainly locate citizens, which could be improved by combining it with different possibilities such as collaborative mapping and the Internet of Things. Adopting a separate approach to cope with distinct hazards and phases of risk management does not match the risk reality, which, as aforementioned, is complex and systemic, with a strong interrelationship between different types of components and actors. In addition, only 28–36% of the pre- and trans-post-event apps allow the sharing of information through other social networks or SMS. This reduces efficiency, making it more difficult to coordinate a humanitarian response, communicate, establish a relationship with forecasting models and spatial–temporal advice and detection systems, and strengthen local coping capacities among regions.

Most apps were developed by private companies with non-transparent information management and ambiguous privacy policies. The results of the analysis highlighted the need for robust policies that promote reliability, transparency, and citizen empowerment and control over their data, which must be provided by a strong public service with a holistic approach.

To evaluate the potential of mobile apps as disaster-mitigating tools regarding natural events, a Digital Application Potential Index (DAPI) was created. The DAPI offers both a general overview and in-depth details on specific topics within its four categories: access to technology, its usage, skills, and affordability at a regional scale. This index has been cross-referenced with the WRI to evaluate the viability of this technology in areas with different rates of disaster risk. A general tendency that could be identified in this analysis lied in the fact that when countries were grouped according to their risk category (based on the WRI), the higher the disaster risk the lower the DAPI. Furthermore, a significant dispersion of data was yielded, albeit with variations between specific DAPI categories. Regarding the DAPI components, one of the main drawbacks uncovered was affordability, which revealed that only countries with low and very low risk of natural event-related disasters maintain affordable prices below 2% of GNI p.c. The mean price for countries with a very high and high disaster risk was 11.3% and nearly 12% of GNI p.c., respectively. The price reduction in the latter together with electricity and broadband access will contribute to a fast increase in mobile phone penetration. In addition, since countries with very high and high disaster risk exhibited a significant number of local and indigenous languages, tools must be adapted to their specific languages and dialects to reach the highest amount of people, especially the most vulnerable. In addition, a clear app layout must be prioritised to reach both the illiterate and the poorest population with lower literacy rates.

The policies to implement the use of mobile apps as tools for Risk Disaster Management must consider reducing the prices of internet connection while increasing educational levels and the language translation of the apps. Their current use falls far short of ensuring communication in Disaster Risk Management and a timely response to natural hazards, especially in countries with a higher level of disaster risk. Other alternatives such as television, radio, and SMS must still be considered.

The index developed has provided interesting recommendations for specific cases, although the same was not true at the global scale, due to the strong divergence of circumstances within each country when these are grouped by risk level. Thus, a detailed study at a regional scale using DAPI is warranted, including each of its components. This would result in more precise recommendations for each geographical area.

Complementarily to this, and as a future line of research, it would be pivotal to evolve from isolated sectoral applications to public digital ecosystem platforms that integrate different tools, levels, and actors for the coordinated operation of integrated disaster risk management.

## Data Availability

Database has been provided.

## Footnotes

1 The deaths by cyclones are not displayed since they are not georeferenced in the international database, as happens with those stemming from floods and earthquakes.

2 The five components are arranged in a circular-spiral model of DRM approach. This facilitates the understanding of disasters as integrated processes in which the stages that occur in the pre-disaster period are linked to those of the post-disaster period. The spiral feature of the model highlights the upgrade in resilience with each passing cycle (Foresti, 2015; Ludwig & MATTEDI, 2018).

3 When missing data, the following adjustments were made for the calculations:

- when an indicator’s component value was unavailable for a specific country, the weight was redistributed among the remaining components;
- the access was not computed if two indicators were not available;
- the overall DAPI value was only obtained when all four components were present.
